# Phase Entropy Analysis of Electrohysterographic Data at the Third Trimester of Human Pregnancy and Active Parturition

**DOI:** 10.3390/e22080798

**Published:** 2020-07-22

**Authors:** José Javier Reyes-Lagos, Adriana Cristina Pliego-Carrillo, Claudia Ivette Ledesma-Ramírez, Miguel Ángel Peña-Castillo, María Teresa García-González, Gustavo Pacheco-López, Juan Carlos Echeverría

**Affiliations:** 1 Faculty of Medicine, Autonomous University of the State of Mexico (UAEMex), Toluca 50180, Mexico; acpliegoc@uaemex.mx (A.C.P.-C.); ciledesmar@uaemex.mx (C.I.L.-R.); 2 Basic Sciences and Engineering Division (CBI), Campus Iztapalapa, Metropolitan Autonomous University (UAM), Mexico City 09340, Mexico; mapc@xanum.uam.mx (M.Á.P.-C.); mtgg@xanum.uam.mx (M.T.G.-G.); 3 Biological and Health Sciences Division (CBS), Campus Lerma, Metropolitan Autonomous University (UAM), Lerma 52005, Mexico; g.pacheco@correo.ler.uam.mx

**Keywords:** electrohysterography, uterine electromyogram, phase entropy, labor, third trimester of pregnancy, nonlinear dynamics

## Abstract

Phase Entropy (*PhEn*) was recently introduced for evaluating the nonlinear features of physiological time series. *PhEn* has been demonstrated to be a robust approach in comparison to other entropy-based methods to achieve this goal. In this context, the present study aimed to analyze the nonlinear features of raw electrohysterogram (EHG) time series collected from women at the third trimester of pregnancy (TT) and later during term active parturition (P) by *PhEn*. We collected 10-min longitudinal transabdominal recordings of 24 low-risk pregnant women at TT (from 35 to 38 weeks of pregnancy) and P (>39 weeks of pregnancy). We computed the second-order difference plots (SODPs) for the TT and P stages, and we evaluated the *PhEn* by modifying the *k* value, a coarse-graining parameter. Our results pointed out that *PhEn* in TT is characterized by a higher likelihood of manifesting nonlinear dynamics compared to the P condition. However, both conditions maintain percentages of nonlinear series higher than 66%. We conclude that the nonlinear features appear to be retained for both stages of pregnancy despite the uterine and cervical reorganization process that occurs in the transition from the third trimester to parturition.

## 1. Introduction

Over the last few years, entropy measures have emerged to study the time series from physiological systems such as the cardiovascular, the muscular, and the nervous system, as well as the female reproductive system [1]. Specifically, a physiological organ like the uterus involves the complex interaction of several subunits or myometrial cells [2]. The analysis of uterine dynamics by entropy-based methods along different pregnancy stages, as well as pathological conditions during pregnancy, has recently become a relevant field of study and research in the clinical, engineering, and physics fields [3,4,5].

The uterine electromyogram or electrohysterogram (EHG) is a non-invasive signal used to study the electrical dynamics of uterine muscles from the abdominal surface in pregnant and nonpregnant women [6,7,8]. The time series of EHG show intermittent bursts of action potentials associated with uterine contractions and basal activity when the uterus is at rest. Interestingly, previous studies indicate that spontaneous uterine contractions contain nonlinear features [9]. Additionally, changes in the dynamics of EHG between term low-risk parturient and non-parturient women have rarely been explored by using diverse entropy approaches [7,10,11]. Authors highlight the necessity of finding suitable measures that characterize electrohysterographic time series generated by the uterine activity during parturition [12].

Phase Entropy (*PhEn*) was recently introduced by Rohila and Sharma for evaluating complexity in physiological signals [13]. *PhEn* quantifies the distribution of the signal in a two-dimensional phase space or second-order difference plot (SODP). Authors found that the *PhEn* showed better discriminative and descriptive power in comparison to other existing entropy measures such as Approximate Entropy (ApEn), Sample Entropy (SampEn), Fuzzy Entropy (FEn), Permutation Entropy (PermEn), Bubble Entropy (BEn), and Grid Density Entropy (GDE) [13]. In comparison with *PhEn*, these other entropy measures failed to identify time irreversibility in time series, a key feature of complex systems [13]. However, the *PhEn* did capture the time irreversibility, and it measured the rate of variability, as well as the multiplicity and compressibility. Thus, Rohila and Sharma concluded that *PhEn* quantifies the complexity of time series [13].

In this study, we proposed the use of *PhEn* to analyze raw EHG time series collected from low-risk pregnant women in the third trimester of pregnancy (TT) (non-parturition) and during active parturition (P). The importance of the present exploratory study is on its theoretical contributions for understanding EHG, specifically in terms of its dynamics, nonlinear features, and reorganization of myometrial fibers in term parturition. In physiological terms, the parturition process implies a high energy expenditure caused primarily by the increasing contractile activity in the myometrium [14]. We thus hypothesized that the *PhEn* in the EHG time series differs between the third trimester of pregnancy and term active parturition owing to the uterine and cervical reorganization processes that occur in the transition from the third trimester to parturition.

## 2. Materials and Methods

### 2.1. Dataset Description

The EHG data for evaluating the proposed *PhEn* analysis-based method were obtained from a dataset of transabdominal recordings of pregnant women collected during two different stages of pregnancy, namely third trimester (TT) and active parturition at term (P) [15]. Transabdominal data were gathered from 24 healthy pregnant participants who attended the Maternal and Child Research Center (CIMIGen) in Mexico City, Mexico. All experimental procedures were approved by the Ethics Commission of the Biological and Health Sciences Division (CBS) at Metropolitan Autonomous University (UAM), Campus Iztapalapa (ref. CAEDCBS.01.2017). All participants gave their informed consent before participating in the study.

The dataset includes 10 min of transabdominal time series from participants studied at the TT of gestation (from 35 to 38 weeks), not showing any clinical manifestation of the initiation of labor. Our dataset also includes 10 min of transabdominal data from the same participants at the active P stage (>39 weeks), showing the occurrence of three or four uterine contractions in 10 min assessed by uterine activity, and presenting no less than 4 cm of cervical dilatation and 50% of cervical effacement. Participants conformed to the following inclusion criteria: young low-risk singleton pregnant women aged 18 to 32 years old, residents of Mexico City or its metropolitan area, without any manifestation of outgoing infection. The Dataset is available at http://hdl.handle.net/20.500.12222/247.

### 2.2. Extraction of Raw EHG Time Series

Our dataset consisted of 24 transabdominal recordings for the TT and 24 for the P stage recorded at 900 Hz from one single channel using a portable maternal-fetal monitor (Monica AN24^®^, Monica Healthcare, Nottingham, UK) [16]. These time series were acquired using disposable electrodes (Ambu^®^ BlueSensor VL, Ambu A/S, Ballerup, Denmark) placed on the abdomen of a pregnant woman. The skin was initially prepared using an alcohol wipe and abrasive skin tape. Then, the electrodes were placed in a diamond arrangement keeping the common electrode 6 cm above the symphysis pubis and locating the active electrode at 10 cm to the right of the umbilicus, and the ground electrode was positioned close to the active electrode. This configuration is standardized by the maternal-fetal monitoring device, Monica AN24 [17].

**Figure 1:**
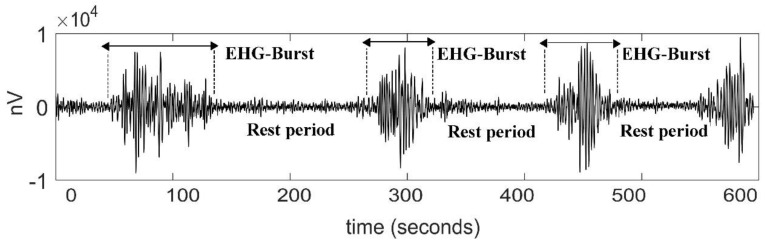
Representative signal of an electrohysterogram (EHG) recorded from the abdominal surface of a pregnant participant in active labor. Uterine contractions are associated with electrical EHG bursts of action potentials.

From these transabdominal recordings, we exported in a text file the extracted raw EHG time series by using the Monica DK software version 1.9 (Monica Healthcare, Nottingham, UK). The software filtered the transabdominal signals in the bandwidth of 0.2 Hz to 1 Hz, which is considered as the dominant frequency band of the EHG [18]. The resulting EHG bipolar signals were downsampled at 20 Hz to reduce the computational cost of the data analysis [19]. According to some authors, focusing on such a range of frequencies allows to exclude most of the artifact components attributable to motion, respiration, and cardiac electrical signals [20]. The time series are expressed in nanovolts (nV) vs. seconds (Figure 1).

### 2.3. Phase Entropy (PhEn)

Phase Entropy (*PhEn*) is an entropy method that quantifies the distribution of the time series in a phase representation [13]. In this study, we followed the methodology proposed by Rohila and Sharma for computing the *PhEn*, but here applied to longitudinal EHG time series of pregnant women in two pregnancy stages, TT and P. First, we created the second-order difference plot (SODP) for the EHG time series corresponding to the TT and P stages. Thus, from a given whole EHG discrete time series EHG[n], we calculated the time-delayed time series Y[n] and X[n] as follows:

(1) Y[n]=EHG[n+2]−EHG[n+1]

(2) X[n]=EHG[n+1]−EHG[n]

**Figure 2:**
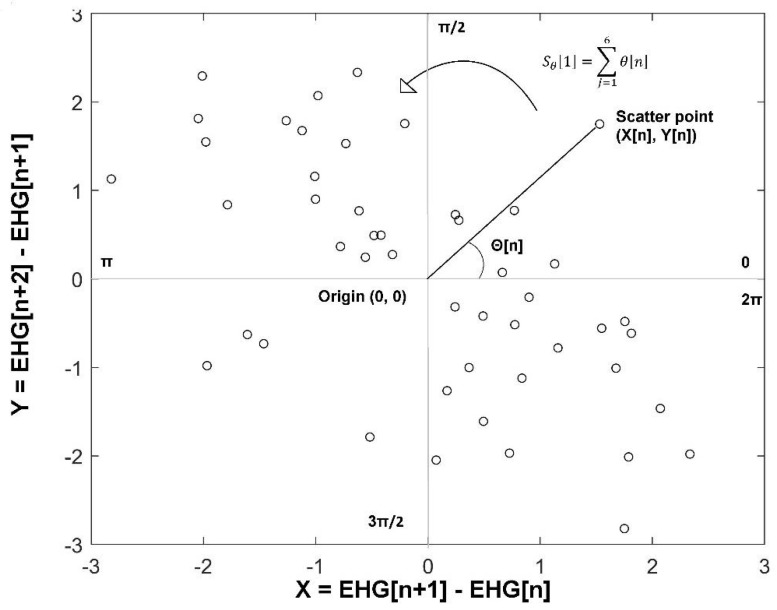
Four-quadrant second-order difference plot (SODP); the phase space from 0 to 2π is split into four sectors (k=4). Scatter points represent corresponding Y[n] against X[n]. In this example, it is shown the calculation of the slope angle (θ[n]) of the scatter point that is connected to the origin (0, 0) in a two-dimensional phase space is a representation of a time series. The partial slope angle of each sector $S_{\theta }\left[i\right]$ is calculated by adding the slope angle of each scatter point within that sector in a counterclockwise way (an example is depicted in the first sector).

Subsequently, we constructed the SODP of EHG as a scatter plot of Y[n] against X[n]. Then, we computed the slope angle of each scatter point (θ[n]) from the origin (0,0), as shown in Figure 2. The entire plot (from 0 to 2π) is split into k sectors having an angle span of $\frac{2\pi }{k}$ radians each. The k value serves as a coarse-graining parameter characterizing how slender a sector is. In Figure 2, a four-quadrant SODP is presented as an example (k=4). In this exploratory study, we set the k value from 2<k<40, because it is a similar range proposed by Rohila and Sharma for exploring the stability of the parameter *k*. According to them, for the computation of *PhEn* it is convenient to select a k value that is divisible by 4 [13].

Next, for each *k*, we obtained the partial slope angle of each sector $S_{\theta }$[*i*] by summing the slope angle of each scatter point within that *i*th sector in a counterclockwise way as follows:

(3) $$S_{\theta }\left[i\right]=\overset{N_{i}}{\underset{j=1}{\sum }}\theta \left[n\right]$$

where *i* = 1,2,3…*k* and *Ni* is the number of scatter points in the *i*th sector.

The probability distribution p(i) of grades in each sector *k* was calculated as follows:

(4) $$p\left(i\right)=\frac{S_{\theta }}{\sum_{j=1}^{i}S_{\theta }}$$

Finally, the *PhEn* is the result of estimating the Shannon entropy computed from the distribution p(i), as shown in Equation (5):

(5) $$PhEn_{k}=\frac{-1}{\log\left(k\right)}\overset{k}{\underset{i=1}{\sum }}p\left(i\right)\log p\left(i\right)$$

The *PhEn* measures the degree of compressibility of the distribution in SODP or the rate of regularity of the time series. Time series that exhibit more richness, variability, and time irreversibility are distributed over all sectors of the plot and are less compressible, thus manifesting higher values of *PhEn* [13]. In terms of the EHG, richness and variability may express the diversity of connections and number of gap junctions in the uterus, respectively [21]. Additionally, a parturient woman, under the response of her regulatory system, may generate an EHG signal with more diverse patterns related to higher uterine bursts of action potentials, perhaps leading to lower compressibility.

All these calculations were done using MATLAB^®^ software version R2019a (MathWorks, Inc., Natick, MA, USA).

### 2.4. Multiscale Entropy (MSE)

To contrast the *PhEn* with other entropy methods, we also calculated the multiscale entropy (MSE) from the TT and P EHG time series. These calculations were performed according to the methodology of our previous study [10]. The MSE curve was obtained over the whole 10-min EHG time series for a range of 1 to 21 scales (*τ*) [22]. Each scale defines the length of the window used for building the coarse-grained time series. The mean value and standard deviation of the SampEn for each scale were obtained for the EHG time series of the TT and P groups. The SampEn value *m* was set to 2 and *r* to 0.15. The MSE is based on the following coarse-grained sequences *y*(*τ*):

(6) $$y_{j}{}^{\left(\tau \right)}=\frac{1}{\tau }\overset{j\tau }{\underset{i=\left(j-1\right)\tau +1}{\sum }}x_{i},\;1\leq y_{j}\leq \frac{N}{\tau }$$

where *τ* represents the scale factor and, for 1 ≤ *j* ≤ *N/τ*, the length of each coarse-grained time series is *N/τ*. For scale 1, the coarse-grained time series is simply the original time series. In addition, *y_j_* is a data point in the newly constructed time series and *x_i_* is a data point in the original time series.

### 2.5. Surrogate Analysis

We performed surrogate data tests on our original EHG time series for TT and P to test the null hypothesis that the underlying process of the EHG corresponds to a stationary linear Gaussian process or uncorrelated noise [23]. Theiler et al. proposed the method of using surrogate techniques to test nonlinearity in time series [24].

To perform these tests, we created 200 phase-randomized surrogates for each original TT or P time series obtained by Iterative Amplitude Adjusted Fourier Transform (iAAFT) with a maximum number of 100 iterations [25]. In this case, the distribution of amplitudes is retained. Phase-randomized surrogates retain the power spectrum and, therefore, the autocorrelation function of the investigated time series. The iAAFT surrogates are constructed to retain both the distribution and the power spectrum as close as possible to the original time series. However, the temporal correlations and potential nonlinear interrelations are removed by randomizing the phases [25].

The iAAFT surrogate series were created, and the *PhEn* was calculated for each of them, with different values of *k* (2 < *k* < 40). Therefore, the linear dynamics null hypothesis was tested individually at any *k* for all TT and P time series. If the *PhEn* at the specific *k* of the original series was lower than the fifth percentile of the distribution of *PhEn* values computed in the entire surrogate set, the null hypothesis of linear dynamics was rejected and the original time series at the specific *k* was deemed to be nonlinear, as suggested by Silva et al. [26].

Finally, for further comparisons, we constructed time-reversed series from the original TT and P time series (rTT and rP, respectively).

### 2.6. Statistical Analysis

The null hypothesis to compare the means values of *PhEn* and MSE for the TT and P time series at different sectors or scales was evaluated by two-way ANOVA with repeated measures (normality was accepted by the Kolmogorov-Smirnov normality test). Data were further analyzed by Bonferroni post hoc test and corrected for multiple planned comparisons. Data were expressed as mean ± SD for homogeneous data visualization.

The Kolmogorov-Smirnov normality test was also applied to test whether the *PhEn* values obtained from the surrogate dataset followed a Gaussian distribution. A chi-squared (χ^2^) test was applied to compare the percentage of nonlinear dynamics derived from the TT and P surrogate time series at different *k*. α = 0.05 was considered as the significance level for all the calculations. Moreover, complementary individual tests for time irreversibility analysis were performed by the non-parametric Wilcoxon signed-rank test (normality was rejected for such time-reversed time series).

All the statistical analysis was carried out using GraphPad Prism version 8.02 for Windows (GraphPad Software, La Jolla, CA, USA).

## 3. Results

**Figure 3:**
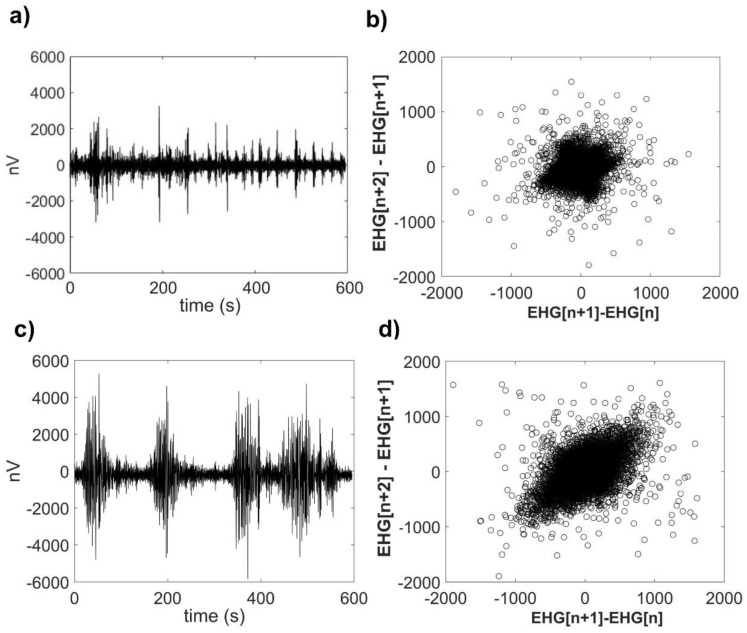
Representative raw electrohysterogram (EHG) time series and their corresponding second-order difference plot (SODP) representations. (**a**) Raw EHG time series from one participant at the third trimester of pregnancy (TT stage, 34 weeks of gestation); (**b**) SODP calculated from this TT raw EHG time series; (**c**) raw EHG time series from the same participant during term active parturition (P, 41 weeks of gestation); and (**d**) SODP calculated from this P raw EHG time series.

A representative raw EHG time series for TT and P conditions from the same participant and their corresponding SODP are shown in Figure 3. Following a visual analysis of this graph, it is possible to note that at TT, the amplitude of the uterine electrical activity is lower (Figure 3a). However, at active P, the amplitude of the uterine activity is larger and shows more variability (Figure 3c). Notably, the SODP of P (Figure 3d) exhibits a wider distribution of scatter points over the whole phase space in comparison to TT, which shows a dense concentration of scatter points around the origin (Figure 3b). While the TT time series was obtained at 34 weeks of gestation, the P time series was derived from transabdominal recordings performed at 41 weeks of gestation.

**Figure 4:**
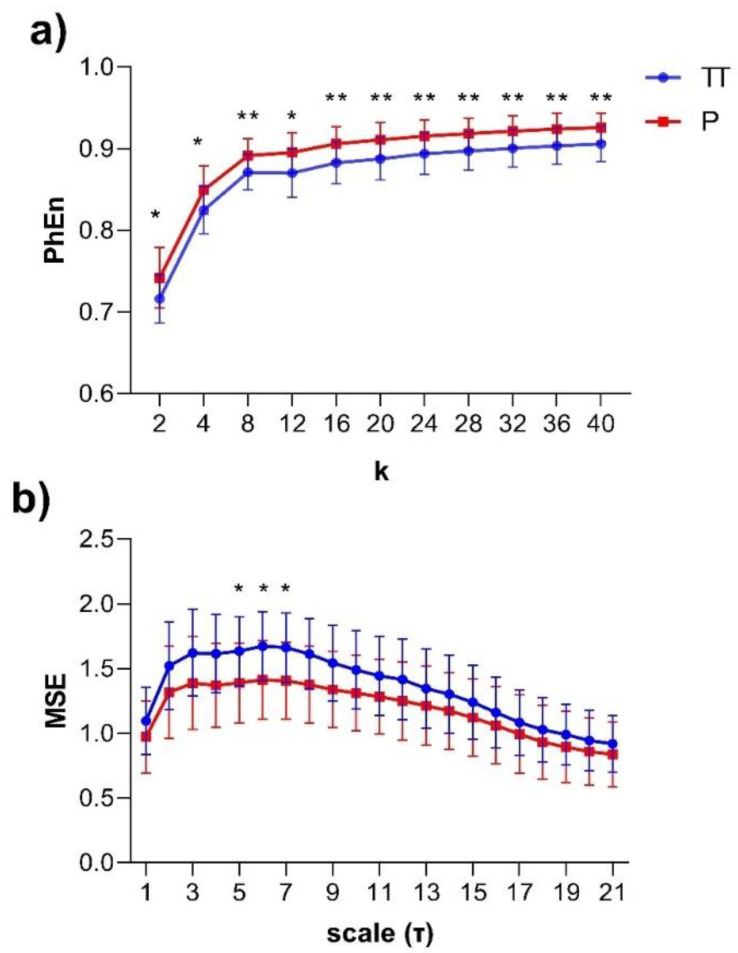
(**a**) Mean values ± SD of the Phase Entropy (*PhEn*) for the range 2<k<40 of the longitudinal electrohysterogram (whole EHG) time series of 24 pregnant women in two different stages: third trimester of pregnancy (TT, continuous blue line, from 35 to 38 weeks of pregnancy) and active parturition at term (P, continuous red line, >39 weeks of pregnancy); (**b**) mean values ± SD of the Multiscale Entropy (MSE) for 21 scales (*τ*) with the Sample Entropy (SampEn) estimator applied to EHG at TT and P. * *p* < 0.05 between TT vs. P and ** *p* < 0.01 between TT vs. P according to the Bonferroni post hoc test.

**Figure 5:**
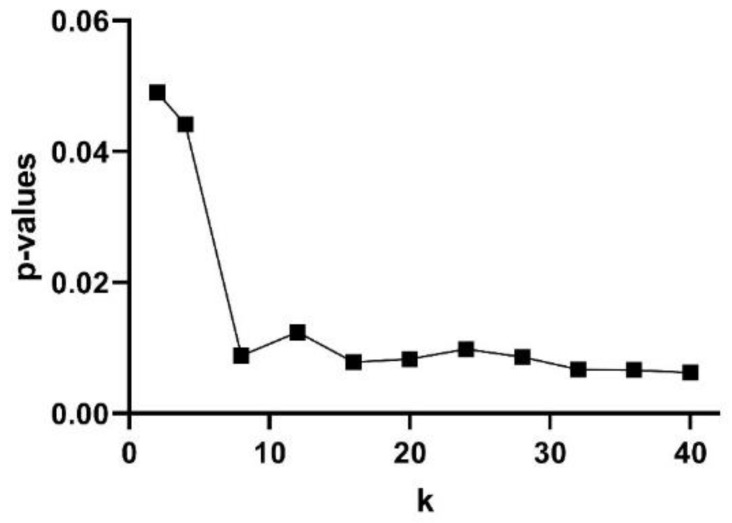
Discriminating power of the Phase Entropy (*PhEn*) at different *k* values: *p*-values according to the Bonferroni post hoc test between the *PhEn* values of the third trimester of pregnancy (TT) and active parturition (P) conditions.

The subjects’ curve profiles (mean ± SD) of *PhEn* for TT (continuous blue line) and P (continuous red line) for the range 2<k<40 are shown in Figure 4a. We found significantly higher mean values of *PhEn* for P compared to TT for all *k* values (*p* < 0.05, Figure 4a). There is a tendency of increasing differences between both conditions with higher k values; lower *p*-values (*p* ≤ 0.007) were found for k≥32 (Figure 5). Additionally, Figure 4b shows the curve profiles calculated by MSE with the SampEn estimator, at TT and P. Contrary to the *PhEn* results, the MSE revealed significantly higher mean values for TT in comparison with P (*p* < 0.05, for scales *τ* = 5–7). This relevant finding seems to confirm that *PhEn* and MSE provide different information. By comparing *p*-values between both entropy methods, *PhEn* seems to be more appropriate than MSE to identify differences between TT and P.

**Figure 6:**
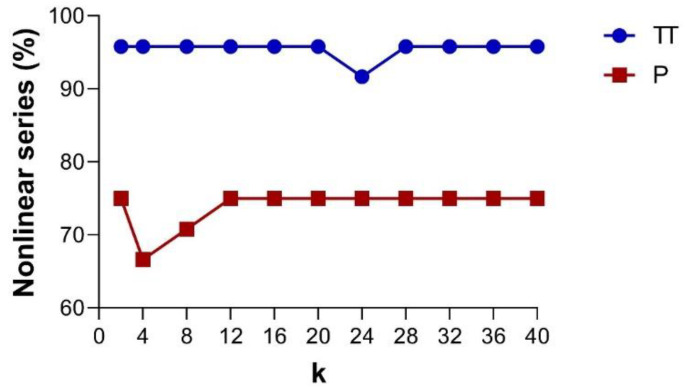
Based on the *PhEn*, the line plots show the percentage of nonlinear series identified by using the EHG time series phase-randomized surrogate test as a function of the *k* value. The graphs show the results for the third trimester of pregnancy (TT, continuous blue line) and active labor at term (P, continuous red line), n = 24.

Based on the *PhEn*, Figure 6 shows the percentage of nonlinear series as a function of the *k* value found in the TT and P conditions, as indicated by the phase-randomized surrogate test [24,25]. The graphs distinguish the percentage of nonlinear dynamics in TT (continuous blue line) and P (continuous red line). From this figure, it is noted that the time series of the TT condition show a larger number of cases exhibiting nonlinear dynamics in comparison with those of the P condition. We observed significant differences (*p* < 0.05) in the number of cases with nonlinear dynamics at any *k* value (except for *k* = 24) between TT and P. Additionally, it is important to note that the percentage of nonlinear series found for both conditions are quite similar at different *k* values.

**Figure 7:**
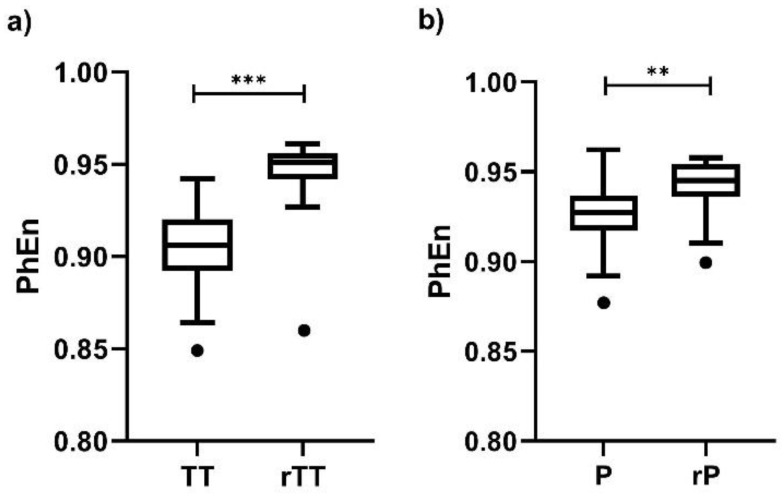
Boxplot of Phase Entropy (*PhEn*) values for k=36 computed from the raw electrohysterogram (EHG) time series of the third trimester of pregnancy (TT) and active parturition (P) and of their corresponding time-reversed versions (rTT and rP, respectively): (**a**) TT vs. rTT and (**b**) P vs. rP. Boxplots show the median, Tukey whiskers (median ± 1.5 times interquartile range), and outliers (●). N = 24, *** *p* < 0.0001, ** *p* < 0.002 obtained by Wilcoxon signed-rank tests.

Finally, we found differences between the time-reversed and the original EHG time series for all k values; the individual result for k=36 is presented in Figure 7. Interestingly, the differences are more discernable in TT vs. rTT (Figure 7a, *p* < 0.0001) than in P vs. rP (Figure 7b, *p* < 0.002), but at both conditions the *PhEn* values are affected by changing the time order of the original time series.

## 4. Discussion

### 4.1. PhEn and MSE

In this study, we evaluated the EHG during active parturition by estimating the *PhEn*; changes were expected in this entropy between the time series of TT and P conditions owing to the uterine and cervical reorganization processes that occur in the transition from the third trimester to parturition. This assumption was supported by the *PhEn* curves obtained in the range of values from 2<k<40 (Figure 4a). The results showed that such a metric has the potential to differentiate the uterine electrical activity of the third trimester (TT) of pregnancy in comparison with that presented during active parturition at term (P), revealing higher *PhEn* in P in comparison to the TT condition (*p* < 0.01).

Previous studies reported that the irregularity of EHG evaluated by SampEn gradually decreases during pregnancy [11]. Other evidence indicates that the values of SampEn are higher during the latent phase in comparison to the active phase of labor, indicating that the irregularity of the uterine EHG signal is reduced in the active phase of labor [27]. Specifically, to compare the *PhEn* results with SampEn, multiscale entropy (MSE) was also applied here to our same dataset. Interestingly, by using the MSE approach, it is likewise possible to discriminate between the uterine electrical activity generated at TT from that generated at P (Figure 4b, *p* < 0.05). This finding is in line with our previous studies, which indicated that the TT time series exhibited more irregular dynamics than P [10]. A possible physiological explanation for our result would be that the periodic components of the uterine contractions at P produce a less irregular behavior.

A system can be considered to be complex if it involves dynamics that are nonlinear, non-stationary, or time-irreversible, and if it shows fluctuations on multiple time scales [28]. Regular time series embrace limited information and can be described concisely with fewer symbols, whereas complex time series are information-rich and therefore are less compressible [29]. Multiplicity is the number of points having the same value in the SODP; it indicates that the distribution is concentrated in a specific sector. Therefore, the probability in such type of sectors increases and the *PhEn* decreases [13].

Authors that introduced the *PhEn* measure indicated that it quantifies some of the nonlinear features, such as multiplicity, rate of variability, time-irreversibility, or even compressibility of time series [13]. Thus, despite the fact that the EHG time series at TT would seem more irregular in comparison with those at P as indicated by MSE, we here provide further evidence revealing that some of those nonlinear features also appear to differ between TT and P. For example, EHG time series at TT are probably more compressible than those at P as suggested by the differences in *PhEn* (while lower values of *PhEn* indicate that the time series are more compressible, higher values reflect less compressibility). This property was visually exhibited in the SODP of TT and P (a representative example is shown in Figure 3b or Figure 3d).

In physiological terms, changes of compressibility, or in other nonlinear features, could be associated with the uterine and cervical reorganization processes that occur in the transition from TT and P [30]. For instance, during the third trimester of pregnancy, the uterus is maintained in a relatively quiescent state with a baseline tone [31]. This basal tone may be related to high redundancies or repetitive patterns in the EHG action potential bursts, producing more compressible time series such as those that we found at TT [32]. However, further studies are necessary to elucidate the specific interpretation of *PhEn* as applied to the EHG time series.

### 4.2. Nonlinearities of EHG Time Series in the Third Trimester (TT) and Active Labor at Term (P)

In the present study, we explored at different *k* values for estimating *PhEn* the potential number of series involving nonlinear dynamics in the TT and P by applying the phase-randomized surrogate test [24,25]. Our findings indicate that the number of time series showing nonlinearity is slightly superior in TT with respect to P (Figure 6). However, both conditions maintain percentages of nonlinear series higher than 66%, suggesting that the nonlinear features are manifested in both TT and P conditions.

These findings seem to be in line with previous evidence concerning uterine contractions, which concluded that the contraction segments could be modeled as a nondeterministic process [33]. Other authors have reported that the nonlinear features of uterine activity are even manifested in nonpregnant women [9]. Radhakrishna et al. indicate that myometrial cells work as oscillators, whose activities are more independent in the initial stages of pregnancy. However, the dynamical coupling between these cells grows stronger as parturition progresses, resulting in the absence of nonrandom structure during uterine contraction [34].

We analyzed the presence of nonlinear dynamics as a function of *k* values; however, the percentage of nonlinear series tends to be constant for diverse values of *k* in both conditions. This result could be associated with the high stability that has been reported by the authors who introduced *PhEn* for different *k* values [13].

According to these authors, another advantage of *PhEn* is that it is sensitive to time irreversibility [13]. Our *PhEn* results of suggest that uterine contractions during TT and P seem to be temporally irreversible as well (Figure 7). This finding is in line with previous results suggesting that the property of time irreversibility is a strong characteristic of uterine contractions [35]. However, P contractions seem to be less time-irreversible than TT, which is consistent with the slightly higher number of series showing nonlinear dynamics that we found in TT.

### 4.3. Future Work and Limitations of the Study

Monitoring contractions during labor is usually performed by external tocodynamometry in the clinical practice; however, this approach can be difficult or even impossible to perform in some patients [36]. In this respect, the EHG has emerged as a new non-invasive potential technique available for monitoring uterine activity. New parameters have been proposed for characterizing the EHG signals; such is the case of *PhEn* assessed in this study. We consider that future studies may involve the utilization of *PhEn* as a potential biomarker for evaluating the physiological onset of human labor or even analyzing preterm labor in clinical practice. Recent relevant studies suggest that nonlinear measures, compressibility in particular, may be useful in improving labor prediction as a complement to other parameters [37]. Additionally, *PhEn* could be incorporated into the current research of pattern recognition [38] of EHG data or even be included in some fetal and maternal analysis software [39,40].

At present, the interpretation and physiological meaning of *PhEn* is diverse. It does not simply measure the degree of compressibility of SODP since the sectors with high angles are privileged. For example, if all points are equally distributed in the SODP, *PhEn* will not be maximum owing to the higher weights attributed to higher sectors. *PhEn* is maximum when *p(i)* is uniform, and it is obtained when sectors become monotonically less populated.

Other limitations imply the inherent complexity of the EHG time series, because complexity differs from contraction to contraction, even in the same labor phase [41].

Given the small sample size in the present study owing to the inherent difficulty of successfully recording longitudinal electrohysterograms along gestation, our findings and interpretation should be confirmed and cautiously taken into consideration. A potential application of *PhEn* could be the detection of preterm labor based on the EHG signal dynamics. Finally, complementary studies should validate *PhEn* models in larger datasets of EHG with different lengths.

## 5. Conclusions

The main findings of this study can be summarized as follows: (1) *PhEn* appears to be more appropriate to differentiate between the third trimester of pregnancy and active parturition in comparison to MSE in response, perhaps, to the uterine and cervical reorganization processes that occur in the transition from the third trimester to parturition. However, both entropy parameters seem to measure different dynamics of the EHG time series at such stages; (2) this study suggests that, by using *PhEn* as the discriminating statistic, the third trimester is slightly characterized by a larger number of series manifesting nonlinear dynamics compared to the parturition condition. Thus, the nonlinear features seem to be retained for both stages of pregnancy. The evaluation of physiological EHG time series by *PhEn* may contribute to obtaining knowledge for assessing uterine dynamics.
